# Exercise effects on functional capacity and quality of life in older patients with colorectal cancer: study protocol for the ECOOL randomized controlled trial

**DOI:** 10.1186/s12877-023-04026-6

**Published:** 2023-05-22

**Authors:** Angela Macías-Valle, Carlos Rodríguez-López, Nicolas María González-Senac, Jennifer Mayordomo-Cava, María Teresa Vidán, María Luisa Cruz-Arnés, Luis Miguel Jiménez-Gómez, Paula Dujovne-Lindenbaum, Maria Elena Pérez-Menéndez, Javier Ortiz-Alonso, Pedro L Valenzuela, Gabriel Rodríguez-Romo, Jose Antonio Serra-Rexach

**Affiliations:** 1 Faculty of Physical Activity and Sport Sciences (INEF), Universidad Politécnica de Madrid, Madrid, Spain; 2 Centro de Investigación Biomédica en Red de Fragilidad y Envejecimiento Saludable (CIBERFES), Madrid, Spain; 3 Department of Geriatrics, Hospital General Universitario Gregorio Marañón. Health Research Institute Gregorio Marañón (IiSGM), Dr.Esquerdo 46, 28007 Madrid, Spain; 4 School of Medicine, Department of Medicine, Universidad Complutense, Madrid, Spain; 5 Colorectal Surgery Unit - General Surgery Department, Hospital General Universitario Gregorio Marañón, Madrid, Spain; 6 Physical Activity and Health Research Group (PaHerg), Research Institute of the Hospital Universitario 12 de Octubre (“Imas12”), Madrid, Spain

**Keywords:** Training, Physical therapy, Colon, Prehabilitation, Rehabilitation, Ageing

## Abstract

**Background:**

Surgery and treatment for colorectal cancer (CRC) in the elderly patient increase the risk of developing post-operative complications, losing functional independence, and worsening health-related quality of life (HRQoL). There is a lack of high-quality randomized controlled trials evaluating the potential benefit of exercise as a countermeasure. The primary aim of this study is to evaluate the effectiveness of a home-based multicomponent exercise program for improving HRQoL and functional capacity in older adults undergoing CRC surgery and treatment.

**Methods:**

This randomized, controlled, observer-blinded, single-center trial aims to randomize 250 patients (>74 years) to either an intervention or a control group (i.e., usual care). The intervention group will perform an individualized home-based multicomponent exercise program with weekly telephone supervision from diagnosis until three months post-surgery. The primary outcomes will be HRQoL (EORTC QLQ-C30; CR29; and ELD14**)** and functional capacity (Barthel Index and Short Physical Performance Battery), which will be assessed at diagnosis, at discharge, and one, three, and six months after surgery. Secondary outcomes will be frailty, physical fitness, physical activity, inspiratory muscle function, sarcopenia and cachexia, anxiety and depression, ambulation ability, surgical complications, and hospital length of stay, readmission and mortality.

**Discussion:**

This study will examine the effects of an exercise program in older patients with CRC across a range of health-related outcomes. Expected findings are improvement in HRQoL and physical functioning. If proven effective, this simple exercise program may be applied in clinical practice to improve CRC care in older patients.

**Trial registration:**

ClinicalTrials.gov ID: NCT05448846.

**Supplementary Information:**

The online version contains supplementary material available at 10.1186/s12877-023-04026-6.

## Background

Colorectal cancer (CRC) is the second most common cancer and the second leading cause of cancer-related mortality in Europe [1]. CRC is more frequent in older than in younger people (31% of affected patients are ≥75 years) [2] and its global prevalence is projected to increase by 2040, especially among older people due to population ageing [3]. Moreover, the expansion of unhealthy lifestyle-related risk factors like westernized diet, obesity, and physical inactivity are contributing to the increase of CRC burden [4].

Tumor resection through surgery is considered the cornerstone of CRC treatment, whereas advanced age is an independent predictor of post-operative mortality and complications [5]. Among older patients undergoing CRC surgery, the presence of geriatric syndromes (e.g., frailty, cognitive and functional impairment) worsens prognosis and increases the risk of developing post-operative complications [6]. Likewise, a recent study has suggested that CRC patients with low muscle mass and muscle quality are more likely to suffer complications after surgery [7]. This is especially relevant in people over 75 years of age, since age-related loss of muscle mass and strength is accentuated [8]. Moreover, the high prevalence of cancer-related cachexia in older people with CRC (approximately 60% in people aged 70 or older) [9] makes this population particularly vulnerable to loss of muscle mass, strength, and physical function (i.e., sarcopenia). Indeed, physical function usually remains below baseline values from three and up to six months following CRC treatment, which could lead to permanent dependency for self-care activities in over half of the older patients [10]. Thus, older patients are at high risk of losing functional independence after CRC treatment.

In older people, a decline in intrinsic capacity (i.e., the composite of all the individual’s physical and mental capacities) increases the risk of functional impairment (i.e., the loss of the individual’s ability to perform activities of daily living (ADLs) independently) and may undermine their health-related quality of life (HRQoL) [11]. In CRC older patients, HRQoL is commonly constructed by disease-specific outcomes (e.g., side effects of surgery and/or radiotherapy, toxicity of systemic therapy, fatigue, pain, bowel functioning) together with other concerns regarding geriatric syndromes and preferences (e.g., functional capacity, social functioning, mood, participation in decision making) [12]. Integration of HRQoL assessment and promotion into care, treatment and follow-up is especially important in older patients with cancer since some of them weight HRQoL over a survival gain compared to younger individuals [13]. Moreover, owing to the improvements in early detection and treatment of CRC, survival has substantially progressed up to 66% at 5 years [14]. Accordingly, there is an increasing number of people living with CRC-related sequelae that threaten their HRQoL [15]. In this regard, the implementation of complementary interventions to anticancer treatments to promote HRQoL and to minimize the consequences of accelerated aging induced by these treatments are warranted [16, 17].

The regular practice of physical activity (PA) in cancer patients has been associated with a better HRQoL [18–23], physical function [21, 24] and functional capacity [18]. PA also contributes to reduce mortality risk [25, 26] and muscle atrophy [27, 28]. In this sense, the World Health Organization [29] and the American Cancer Society [30] recommend performing at least 150-300 minutes per week of moderate-intensity PA or 75-150 minutes per week of vigorous-intensity PA (or a combination thereof) involving resistance and endurance exercise – as well as minimizing sedentary behaviors – for primary and tertiary cancer prevention in adults. Moreover, the benefits of physical exercise as an intervention for promoting healthy ageing and treating age-related disorders in older patients with cancer are well documented [20, 21]. However, there is high inter-individual variability on the effects of exercise interventions that can be explained by several factors such as type of cancer, participants’ or interventions’ characteristics, or type of study. Studies on exercise effects in elderly patients with CRC are scarce and methodologically heterogeneous. A recent review [5] which included 11 randomized controlled trials (RCT) concluded that exercise interventions improve functional capacity [31–33] and reduce postoperative complications [34, 35] and length of hospital stay (LOS) [35, 36] in CRC patients with surgical indication over the age of 60 years. Only two RCTs assessed mortality, but no decrease was found in 30-day [36] or 1-year mortality [35] after the intervention. No differences regarding HRQoL were found [32, 37]. However, there is a lack of consensus regarding the most effective exercise type [38], the optimal dose of exercise [39] or the best moment to implement the exercise intervention (prehabilitation vs. rehabilitation) [40, 41]. In this regard, the combination of exercise with other interventions such as nutritional or mental support and behavior modification before surgery (i.e., multimodal prehabilitation) may not provide additional benefits to multimodal rehabilitation, as showed in a recent Cochrane Review [41]. However, the authors concluded that the low number of included participants and studies (only 250 patients among three RCTs) summed to their methodological heterogeneity might have limited the certainty of their conclusions so more and larger studies are needed to gather evidence. On the other hand, adherence to these programs is often inconsistently reported, and there are no clear recommendations on how to increase it [42]. Previous studies have shown that enrollment in exercise trials and adherence to exercise is negatively associated with older age [43, 44]. Some commonly reported barriers include physical symptoms related to cancer or its treatment (e.g. fatigue, peripheral neuropathy, comorbid health conditions, impaired mobility), psychological impact of cancer diagnosis, many hospital appointments, lack of information, lack of social support, and other constrains regarding time, costs, access and guidance [44, 45]. Low-cost and easily accessible programs that can be carried out at home and that are supported and guided by professionals (via direct supervision, written materials or by phone) may improve adherence to exercise [44, 46]. Therefore, the design of these programs should be adapted according to the characteristics of the population studied, the patient’s individual level of performance, the proposed outcomes and patient’s preferences [5, 47].

There is still a lack of knowledge on the benefits of exercise in older and more vulnerable patients, who are precisely those with the highest risk of complications and the greater loss of functional independence and HRQoL after CRC treatment. For this reason, we aim to study the effects of an individualized home-based multicomponent exercise program, supervised by a clinical exercise physiologist through telephone calls, on functional capacity and HRQoL in older adults undergoing elective CRC surgery when compared to usual care. Our primary hypothesis is that the exercise program improves functional capacity and HRQoL of older patients with CRC compared to usual care. The secondary aims will be to investigate the effect of the exercise program on physical performance, surgical complications and hospital readmissions, the prevalence of frailty, anxiety and depression, sarcopenia and cancer-cachexia, as well as the effects on physical fitness and inspiratory muscle function in comparison to usual care. Furthermore, the intervention’s safety and feasibility will be studied by registering patient’s adverse events and adherence to the program.

## Methods

### Study design and ethical approval

This study is a non-pharmacological, randomized, parallel-controlled, observer-blinded trial organized by the Health Research Institute Gregorio Marañón of Madrid, Spain. The study design, protocols and informed consent procedures were approved by the Medical Ethics Committee of *Hospital General Universitario Gregorio Marañón* (HGUGM) *(Madrid, Spain*) (ref.18/2021) and registered in clinicaltrials.gov (NCT05448846). The present study protocol will be conducted following the SPIRIT (Standard Protocol Items: Recommendations for Interventional Trials) statement and in accordance with the ethical guidelines of the Declaration of Helsinki [48]. The study will be carried out in the health care area of the HGUGM (Madrid, Spain) over a 3-year period.

### Study participants, screening and recruitment

**Fig. 1:**
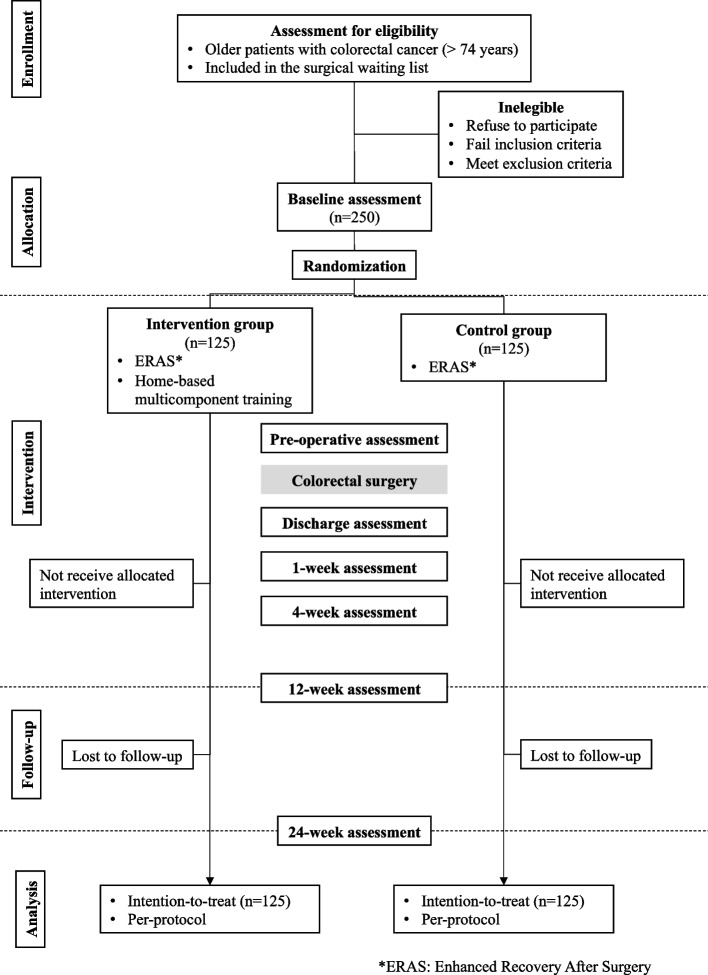
Flow diagram of the study

Patients will be screened, informed, and included in the study at the HGUGM, Madrid, Spain. All patients will be provided a written informed consent. CRC patients aged 75 or older will be recruited at the geriatric outpatient clinic after the completion of a comprehensive geriatric assessment (CGA). The trial inclusion criteria will be: I) being 75 years or older, II) confirmed CRC diagnosis, III) being included in the surgical waiting list, and IV) being able to communicate, and able and willing to provide informed consent. The trial exclusion criteria will be: I) dismissal for surgery, II) any active limitation which affects adherence to the study procedures like terminal disease, myocardial infarction, or fractures in any limb (within the last three months before study participation), inability to walk or severe dementia (i.e., scoring Mini-Mental State Examination ≤ 18 points). Patients will be previously informed about the program’s content, study aims and randomization and about the role of care providers and research staff during participation. An overview of the study participant’s flow diagram is shown in Fig. 1.

### Randomization and blinding

Patients who fulfill the inclusion criteria and accept to participate will be randomly assigned (1:1) to an intervention (IG) or control group (CG) following a computer-generated (Sealed Envelope Ltd. 2021) allocation sequence using a fixed block size of 6. The allocation sequence and resulting patient assignment will be concealed from study members involved in participants’ enrollment and assessment. Patients will be notified about allocation consignment by the study member involved in the intervention delivery, who will advise to participants not to discuss about their treatment assignment with blinded staff. Participants and care providers will be not blinded to the treatment group. By contrast, data collection and analysis will be performed by researchers blinded to the group allocation of the patients.

## Interventions

### Control group (CG)

Participants randomly assigned to the control group will receive the usual care, which includes the Enhanced Recovery for Abdominal Surgery (ERAS) by the surgery department [49, 50]. This includes oral and written information on diet, avoiding sedentary lifestyle and breathing exercises with a spirometer, among other items that try to assure an early post-operative recovery. The participation of the geriatric team includes the CGA-guided intervention, mostly focused on the management of frailty and other geriatric syndromes, such as malnutrition, functional impairment, cognitive decline or depression and quality of life. In addition, the clinical exercise physiologist will give participants general advice about the positive effects of PA and will encourage them to reduce sedentary lifestyle.

### Intervention group (IG)

In addition to usual care received by the CG, patients in the IG will complete an individualized home-based multicomponent exercise program, supervised through telephone calls by a clinical exercise physiologist specialized in dealing with older patients, who is part of the local care team.

#### Exercise intervention

The Consensus on Exercise Reporting Template (CERT) is followed to provide elemental information about the exercise intervention [51]. The exercise intervention will be separated into I) pre-operative phase (prehabilitation), lasting from patient’s enrollment to hospital admission for CRC surgery, II) perioperative phase, from CRC surgery to patient’s discharge from hospital, and III) post-operative phase (rehabilitation), extending up to 12 weeks after CRC surgery. According to leading guidelines on exercise prescription for older adults [52], the exercise program will be tailored to the functional capacity of the patients. Thus, patients will be classified into 4 different groups depending on their Short Physical Performance Battery (SPPB) score, as follows: I) person with disability (SPPB score 0 to 3); II) frail person (SPPB score 4 to 6); III) pre-frail person (SPPB score 7 to 9); and IV) robust person (SPPB score 10 to 12) (see below: *Physical Performance*). The SPPB score will be the decision rule for determining the starting level of the program, which will be reviewed at subsequent face-to-face training sessions by the clinical exercise physiologist. A total of three face-to-face training sessions will be conducted individually at baseline (at own home for adjusting program to the environment of the patient), during hospitalization (once early mobilization of the patient is indicated), and one month after CRC surgery in the hospital setting. In these face-to-face training sessions, the clinical exercise physiologist will provide the corresponding education materials, guidance and motivation to complete the exercise program, observe exercise performance, correct exercise technique, and give feedback to the patients. The educational materials include an informative notebook with the explanations of the exercise program, an exercise diary and audiovisual material. Moreover, patients will receive instructions on how to complete the exercise diary for recording exercise sessions (e.g., exercise modality, repetitions, duration, intensity) of each training session.

The home-based multicomponent exercise program will involve 2 weekly training sessions aimed at improving strength and balance (resistance training) and 2 weekly training sessions aimed at improving cardiorespiratory fitness through brisk walking (endurance training), which will be complemented with daily inspiratory muscle training (IMT). Patients will be asked to complete resistance and endurance training sessions on alternate days, scheduled along the week according to their preferences.

#### Resistance training

Resistance training sessions will involve a progressive combination of activities including: I) mobilization and warm-up (range of motion exercises for the neck, shoulders, arms, trunk, and legs); II) a lower-body strength exercise (sit-to-stand); III) an upper-body strength exercise (seated curl to press exercise with a free-weight load like a pair of water bottles); IV) balance (one-leg standing balance); and V) calm down (stretching and relaxing exercises). Additional file 1 shows this in more detail. During hospital stay, a supervised face-to-face session will take place for the adequacy of the post-surgery exercise. Following the advice of our surgical team, the lower-body strength exercise (i.e., sit-to-stand exercise) will be replaced by a seated knee extension exercise in order to avoid excessive intra-abdominal pressure during the 4 weeks following surgery [see Additional file 2]. Overall, patients will be advised to complete all movements in a slow and controlled fashion during the first two weeks of the program and the 4 weeks after surgery. Then, patients will progress to complete the concentric phase (muscle shortening) as fast as possible, followed by a controlled, slower eccentric phase (muscle lengthening) to maximize mechanical power output and motor unit recruitment (i.e., power training) [53]. Besides, patients will be trained to avoid Valsalva's maneuver during exercise execution.

#### Endurance training

Endurance training sessions will consist of brisk walking (i.e., walking faster than habitual gait speed). Patients will be instructed to walk at a pace that hinders them from talking comfortably, but that allows them to maintain a conversation (i.e., the equivocal stage of the talk test) [54]. Several studies have suggested that the equivocal stage of the talk test is a valid indicator of the ventilatory threshold [55]. Patients will walk independently or assisted (e.g., with the help of other person, a walker, or a walking stick). Overall, endurance training sessions will aim to accumulate at least 30 minutes of continuous or interval brisk walking.

#### Inspiratory muscle training (IMT)

A specific device for IMT (POWERbreathe Medic Plus, Southam, United Kingdom) will be used. Patients will complete 30 repetitions twice a day with a starting load (i.e., intensity) corresponding to the 40% of patient’s maximal inspiratory pressure (PI_max_, see inspiratory muscle function section below for more details), measured at baseline and 1, 3, and 6 months after surgery. For every repetition, the patients will be instructed to exhale until achieving residual volume and will then perform a maximal inspiration (i.e., as fast and for as long as possible) through the IMT device. If needed, patients will be allowed to include a brief rest (30 to 60 seconds) every 5 to 10 repetitions. IMT sessions will be completed twice a day, once after the corresponding resistance or endurance training session, when applicable.

#### Exercise progression

The exercise program will be continuously adapted to the patient’s clinical status and functional capacity. Exercise progression regarding resistance and balance exercises will be achieved by adjusting the number of repetitions, the number of sets, the usage of additional weights and/or by exercise selection (i.e., moving from simple to more advanced exercises). Patients will rate each exercise intensity and overall training session intensity through the Borg’s Perceived Exertion Category-Ratio Scale 1-10 (RPE_1-10_) for aerobic exercise and the OMNI-Scale for resistance training [56, 57]. After a conditioning phase, resistance training prescription will be tuned weekly to target an intensity ranging between moderate to vigorous (i.e., 5- “strong”- to 7- “very strong”- points RPE_1-10,_ respectively). For guidance, if the patient complies with the exercise prescription, the training volume will be increased (i.e., higher number of repetitions and/or sets) or a more complex exercise will be selected based on the established criteria (Table 1). In contrast, scoring more than 8 points RPE_1-10_ will be considered a regression criterion, which entails a decrease of 2-3 repetitions per exercise and/or selecting an easier exercise. On the other hand, endurance exercise will range from 10 minutes (starting level of group I of disabled patients) to 60 minutes per session, while exercise progression will be set according to the establish criteria (Table 1). Accordingly, if the RPE_1-10_ for endurance training session exceeds 8 points, session duration will be reduced by 5 minutes. In terms of IMT, the patients will be instructed to increase the load of the threshold device between 0.5 and 1 levels (i.e., 3.5 and 7 cm H_2_O) every week if being able to complete twice a day 30 repetitions. If return to exercise/usual activities is delayed due to post-operative complications, study participants will continue in the study and restart the exercise intervention once they are able until completing the 12 weeks of training.

**Table 1:** Training regression and progression criteria

**Starting level (SPPB score)**	**RESISTANCE TRAINING**						**ENDURANCE TRAINING**
	Lower body		Upper body		Balance		Brisk walking
	**Sets**	**Repetitions**	**Sets**	**Repetitions**	**Sets**	**Time (s)**	**Time (min)**
**A (0-3)**	1 - 3	3 - 5	1 - 3	3 - 5	3	3 - 5	10 - 15
**B (4-6)**	2 - 3	5 - 8	2 - 3	5 - 8	3	3 - 5	15 - 20
**C (7-9)**	2 - 3	8 - 10	2 - 3	10 - 15	3	3 - 5	20 - 25
**D (10-12)**	3	10 - 12	3	10 - 12	3	5 - 10	25 - 30
**E (12)**	3	10 - 15	3	10 - 15	3	10 - 15	≥ 30

*SPPB* Short Physical Performance Battery

#### Adherence and exercise monitoring

Patients will be contacted weekly on a 20-minute telephone support session by the clinical exercise physiologist to report adherence to the home-based training. Thus, every week they will receive an explanation about the purpose of the intervention and the possible benefits of the exercises and progress will be reviewed. Motivation strategies will be used for achieving engagement to sustain exercise activity and/or achieve higher or progressively more intense performance. These strategies are based on the principles of Self Determination Theory [58]. Participants will have the opportunity to discuss any issues related to their exercise program and any barriers to exercise will be taking into consideration. Attendance of each participant will be recorded and reasons for dropouts will be documented.

### Primary outcomes

#### Health-related quality of life

HRQoL will be assessed with the European Organization for the Research and Treatment of Cancer Quality of Life Questionnaire Core 30 (EORTC QLQ-C30) [59]. The questionnaire includes items for the assessment of the global health status, physical, role, cognitive, emotional, and social functioning, as well as assessment items of symptoms and financial difficulties due to cancer and its treatments. Items are assessed from 1 to 4 (1: not at all, 2: a little, 3: quite a bit, 4: a lot) and overall health and the patient's quality of life from 1 to 7 (1 for very poor and 7 for excellent). The total score ranges from 0 to 100 (higher score is considered better). A change of 10 points or more is considered clinically relevant [60]. As complementary questionnaires we will also use the EORTC QLQ-CR29 [61], which includes 29 items specifically aimed at patients with CRC and the EORTC QLQ-ELD14 [62, 63], aimed at elderly patients (≥70 years) with cancer and that includes 14 items.

#### Functional capacity

The Barthel Index [64] will be used to assess the capacity of patients to execute ten basic activities of daily living (i.e., feeding, transferring from bed to chair, using the toilet, bathing/showering, personal hygiene, dressing, walking, stair climbing, and bowel and bladder control). The total score ranges from 0 (total dependence) to 100 (independence) with intervals of 5 points. A change of 5 or more points is considered clinically relevant [65].

### Secondary outcome measures

#### Physical performance

Physical performance will be measured with the SPPB [66], which assesses the patients’ ability to perform three physical tasks: I) static balance, standing for 10 seconds with their feet together (1 point), in semi-tandem, (1 point) and in tandem position (up to 2 points), II) 4-meter habitual gait speed (0 to 4 points), and III) the ability to stand up from a chair during a five-repetition sit-to-stand test (0 to 4 points). The total score ranges from 0 (worst) to 12 (best). Scoring less than 10 points indicates a high risk of frailty, deterioration, and falls [67]. A one-point change in the test is considered clinically relevant [68].

#### Frailty

The presence of frailty will be assessed according to Fried’s frailty phenotype criteria [69], which includes five health-related domains: involuntary weight loss (> 4.5 kg in the last year), weakness (handgrip strength, JAMAR hydraulic hand dynamometer, Nottinghamshire, UK), self-reported exhaustion during the last week, slowness (4.5-meter habitual gait speed test), and low levels of PA (walking less than 2.5 and 2 hours, or an equivalent energy expenditure, for men and women, respectively). Depending on the overall score obtained, participants will be classified into three different categories: frail (≥3 criteria), pre-frail (1 or 2 criteria) or robust (does not meet any criteria).

#### Physical fitness

Patients’ physical fitness will be evaluated through the Senior Fitness Test, a battery of tests designed for the community-dwelling elderly population [70]. Specifically, this battery assesses the patients’ cardiorespiratory capacity (2-minute step test), strength and flexibility of the lower limbs (30-second sit-to-stand test and chair sit and reach test, respectively) and upper limbs (30-second arm curl test and back scratch test, respectively), balance (Flamingo test), maximal gait speed (10-meter maximal walking test) and agility (Timed-up and go test). A detailed description of each test is available elsewhere [70]. The patient’s score for each capacity will be determined according to the specific normative values (i.e., percentiles) for non-institutionalized Spanish older adults [71].

#### Physical activity

Changes in PA will be determined through the Physical Activity Scale for the Elderly (PASE) questionnaire [72]. Briefly, this questionnaire evaluates the level of self-reported physical activity including occupational, household and leisure activities over the past 7 days in individuals aged 65 years and older. The total PA is computed on an overall weighted score (from 0 to more than 400 points) where the minimal clinically important difference for cancer patients ranges between 17 and 25 points [73]. Current evidence supports the PASE as the best questionnaire for measuring moderate to vigorous PA in older adults [74].

#### Sarcopenia and cachexia

Changes in muscle mass and quality will be recorded by ultrasound examination (Lumify system and L12-4 linear array transducer, Philips Ultrasound, WA, USA) of the lower and upper limbs. Patients will lie on an examination table in supine position with a foam roller placed beneath the knees (i.e., with slight knee flexion). After allowing 10 minutes for fluid stabilization, three cross-sectional images of the rectus femoris (at 50% of the distance between the anterior superior iliac spine and the proximal border of the patella) and biceps brachii muscles (at 66% of the distance between the acromion process and the elbow crease) will be acquired to determine muscle thickness and echo-intensity (i.e., grayscale quantification). In addition, three longitudinal images of the vastus lateralis muscle (at 65% of the distance between the greater trochanter and the distal boundary of lateral femur condyle) with the probe aligned with the fascicle plane will be acquired to determine muscle thickness and architecture (i.e., fascicle length and pennation angle). The image acquisition location and skin landmarks will be transferred to an acetate template to improve measurement reliability of the following visits. All images will be subsequently analyzed using Fiji image analysis software [75] and the Simple Muscle Architecture Analysis tool for Fiji [76]. The determination of low levels of muscle mass, necessary for the diagnosis of sarcopenia, will be determined using the cut-off points for the Ultrasound Sarcopenia Index, which is obtained by the ratio between fascicle length and muscle thickness [77]. The operational definition of sarcopenia revised by the European Working Group on Sarcopenia in Older People and the corresponding cut-off points will be applied for the diagnosis of sarcopenia [78]. In addition, the presence of cancer-cachexia will be evaluated according to the international consensus for the definition and classification of cachexia associated with cancer [79].

#### Inspiratory muscle function

Inspiratory muscle function will be determined measuring the maximum static inspiratory pressure that the patient can generate at the mouth (i.e., PI_max_) against an occluded system (i.e., Müller’s maneuver) (Micro Medical/CareFusion, Kent, UK) [80, 81]. Properly seated, patients will be instructed to exhale up to their residual volume and will be then encouraged to inspire as fast and strong as possible for 3 seconds. The highest 1-second average value will be recorded in each trial. The best three reliable measurements (i.e., < 10% coefficient of variation) will be obtained from a range of 5 to 8 maximum trials separated by 60 seconds of rest.

#### Anxiety and depression

The degree of anxiety and depression will be determined according to the Hospital Anxiety and Depression Scale (HADS)[82]. This is a questionnaire that includes 14 items in two subscales: 7 items to assess anxiety and another 7 to assess depression. Each item can be scored from 0 to 3 points, so the possible total scores range between 0 and 21 for each subscale. A total score from 0 to 7 implies the absence of anxiety and/or depression, from 8 to 10 suggests the presence of a disorder, and a score of more than 11 indicates a probable presence of a mood disorder [83].

#### Ambulation ability

Ambulatory capacity will be measured with the Functional Ambulation Classification (FAC) scale [84]. This scale assesses the patients’ walking ability on 6 levels according to whether they: cannot walk (level 0); walk with continuous manual contact from one person to maintain balance or to assist coordination (level 1); require light physical contact from a person (level 2); walk alone but require supervision of one person either for safety or verbal cueing (level 3); walk independently on level surface, but cannot negotiate stairs, inclines or non-level surfaces (level 4); or walk independently, including stairs (level 5).

#### Surgical complications

Surgical complications during hospital stay will be objectively classified according to the Clavien-Dindo scale [85]. Different categories (from I to V) are defined on this scale based on the severity of the treatments required by patients after undergoing surgery. Thus, categories I-II are considered as mild and III-V as serious.

#### Hospital length of stay, readmissions, and mortality

The LOS associated to CRC surgery will be computed as the number of days from hospital admission for surgery to discharge. The number of hospital readmissions, the length of stay of these readmissions as well as the incidence of all-cause mortality will be registered thought the study.

#### Other variables

Other variables will be collected through a standard questionnaire administered by duly trained personnel. Include: sociodemographic data, clinical variables, medications, comorbidities [86], characteristics of cancer, American Society of Anesthesiologists (ASA) physical status classification to help predict operative risk [87], type of surgery, adjuvant treatment, presence of geriatric syndromes and medical complications.

### Adverse events and patient-reported outcomes

Patients’ adverse events will be registered during the study duration by the medical staff according to the NCI- Common Terminology Criteria for Adverse Events (CTCAEv5.0). In addition, patient-reported outcomes on cancer treatment derived symptomatic toxicities will be collected weekly through a custom-built survey designed at PRO-CTCAE ^®^ Measurement System (https://healthcaredelivery.cancer.gov/pro-ctcae/). The exercise-related adverse events (i.e., those deemed to be caused by exercise) will be captured by the clinical exercise physiologist during weekly phone monitoring. Exercise-related adverse events will be categorized as musculoskeletal/connective tissue (e.g., delayed onset muscle soreness, strains, joint pain), accidents/injuries (e.g., falls, sprain, tripping), cardiovascular symptoms/conditions (e.g., lightheadedness, dizziness, fainting, and heart palpitations), surgery-related (e.g., herniation, stoma leakage, scar tissue pain) or others, as proposed elsewhere [88].

### Data collection and access

Research variables will be collected at baseline, at hospital discharge, and 1, 3 and 6 months after discharge, with the exception of physical fitness at hospital discharge due to medical contraindication. To discern the effect of the intervention during the pre-surgery and hospitalization period, the presence of frailty, ultrasound examination and inspiratory muscle’s function will be recorded at hospital admission prior to surgery (Table 2). Study data will be collected and managed using REDCap electronic data capture tools hosted at HGUGM [89]. Data validation strategies will be applied to avoid problems with manual data entry. All investigators will be giving access to the cleaned data sets according to the role given within the study.

**Table 2:**
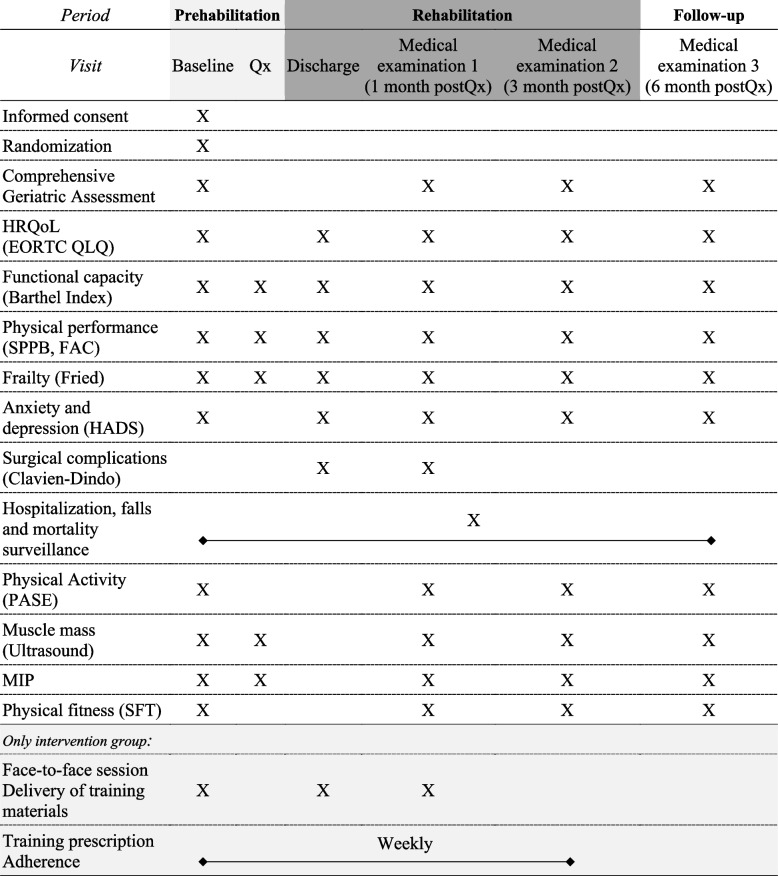
Data collection schedule for ECOOL-Program

*Qx* Colorectal surgery, *HRQoL* Health-Related Quality of Life, *EORTC QLQ* European Organization for the Research and Treatment of Cancer Quality of Life Questionnaires, *SPPB* Short Physical Performance Battery, *FAC* Functional Ambulation Classification Scale, *HADS* Hospital Anxiety and Depression Scale, *PASE* Physical Activity Scale for the Elderly, *MIP* Maximum Inspiratory Pressure, *SFT* Senior Fitness Test

### Sample size and statistical power

A priori sample size is calculated by estimating the difference between groups in the change in the quality scale of life (EORT QLQ-C30) from diagnosis to 3 months after discharge. A systematic review with meta-analysis pooling 25 clinical trials with a total of 547 cancer patients older than 70 years reported a mean ± standard deviation for global health status of 57.4 ± 23 points in the EORT QLQ-C30 scale [90]. If a difference of 10 points on this scale is considered clinically significant [60], using a two-sided sample t-test at the 5% level of significance with a statistical power (1-β) of 80%, we need to analyze a total of 166 patients. Mortality rate for patients over 74 years of age undergoing CRC surgery at the HGUGM is 4% (unpublished data). Assuming a conservative one-year mortality rate of 20% and an additional drop-out rate of 25% of the patients, we will need around 250 patients (125 per group). Considering a standard deviation of 16.3 points in the Barthel index based on data collected during the CGA for CRC patients conducted during the usual clinical practice at the HGUGM, this sample will be suitable for detecting a 10-points difference between groups, which is larger than the minimal clinically important difference for a geriatric population [91].

### Statistical analysis

Baseline characteristics of included participants will be analyzed after the total sample have completed the first visit. Descriptive data will be stratified by group allocation and sex. Unless otherwise stated, continuous variables will be presented as mean and standard deviation or median and interquartile range, as required. Categorical variables will be presented as absolute and relative frequencies. Groups will be compared at baseline using Student independent t tests or χ^2^ tests for continuous or dichotomous variables, respectively. For primary and secondary outcomes analyses of continuous variables, the changes observed along repeated measurements will be compared within- and between-group using linear mixed model procedures. Group allocation (IG vs. CG) and repeated measurements (i.e., baseline, hospital admission-discharge and 1-month, 3-month and 6-month after surgery) will be included in the model as fixed factors (i.e., independent categorical variables) while random intercepts will be considered for patients to account for within-subject correlation between repeated measurements. Analyses will be adjusted for potential confounders based on variables with significant between-group differences at baseline or previously described factors affecting the corresponding dependent variable. The models will be calculated considering maximum likelihood estimation and the best-fitting covariance structure. The consistency of the between-group differences will be assessed for subgroups defined by age, frailty, presence of delirium, and baseline functional status. In addition, the proportion of patients who loss, maintain or regain HRQoL, functional and physical capacity baseline values at 3 and 6 months after surgery will be compared by using χ^2^. The effect of the intervention on mortality incidence will be analyzed with Kaplan-Meir curves, Log-rank test and Cox regression analysis. Primary analyses will be conducted based on the intention-to-treat principle. Missing data will be addressed with multiple imputation methods while sensitivity analyses will be employed to evaluate the influence of the multiple imputations. The statistical level of significance will be set at α=0.05.

## Discussion

This study aims to determine whether an individualized home-based exercise program supervised by telephone improves or maintains HRQoL and functional capacity in patients ≥ 75 years old during CRC treatment when compared with usual care.

Our main hypothesis is that this individualized home-based exercise program supervised by telephone will improve the HRQoL and functional capacity of older adults following CRC treatment. Although exercise interventions are highly recommended as they provide numerous benefits to people affected by cancer in general, studies including very old populations (i.e., aged 75 years and over) are practically null. Several RCTs on CRC patients aged over 60 years have reported that regular PA improves functional capacity [31–33], reduces postoperative complications [34, 35] and LOS [35, 36]. In contrast, other studies have reported no reduction of 30-day [36] or 1-year mortality [35], nor improvements in HRQoL [32, 37].

As recommended by international guidelines for primary and tertiary cancer prevention in adults [30, 92, 93], most studies include a combination of strength and endurance training. Moreover, most studies include supervised training sessions performed in hospitals or sport centers. Considering that our patients are very old, and adherence is reduced with older age, the inclusion of home-based exercise sessions supervised weekly by phone calls may be an alternative, particularly when accessibility issues, lack of mobility or time constraints are barriers to participate. Nevertheless, scarce information exists regarding the most effective way of training and how to improve adherence to the exercise program for promoting healthy ageing and treating age-related disorders in older people during and after CRC.

To the best of our knowledge, this will be the first RCT conducted in a very old population (over 74 years) of CRC patients, who indeed present more physical, mental, social and emotional limitations and who have an increased risk of suffering the negative consequences of surgery compared with younger populations. Another novelty of our study is that our exercise program will also include inspiratory muscle training unlike other multicomponent programs.

Considering that our sample will be composed of patients who are older than those usually included in research, there may be more declination to participation in the program, more difficulties following and reporting the exercise program, as well as a lower adherence. The best results for improving adherence and motivation to exercise can be expected by providing a structured exercise program with individually tailored dosages based on the patients’ performance, surgical conditions and preferences, as well as with a continuous supervision by telephone [94]. The ECOOL-Program addresses these issues and offers a simple home-based exercise program supervised weekly by telephone which requires low resources and could be easily implemented in the clinical setting. Due to the increasing life expectancy and the growing prevalence of CRC in older adults, there is a need for improving CRC care. We hypothesize that an exercise training intervention applied both before and after CRC surgery may have additional benefits for improving HRQoL and physical functioning in very old patients compared to usual care.

## Supplementary Information

**Additional file 1.** 

**Additional file 2.** 

## Data Availability

The datasets generated during the present study can be obtained from the corresponding author on reasonable request. The trial results will be communicated via publications.
