# Supplementary material for: Exercise effects on functional capacity and quality of life in older patients with colorectal cancer: study protocol for the ECOOL randomized controlled trial
Source: BMC Geriatr. 2023 May 22;23:314. doi: 10.1186/s12877-023-04026-6 (PMC10201762; doi:10.1186/s12877-023-04026-6)
Supplement: Supplementary file 1 — Additional file 1. [file 12877_2023_4026_MOESM1_ESM.pdf]

Additional file 1

ECOOL-Program INTERVENTION

Classification of the groups according to the degree of frailty:

- Person with disability (SPPB 0-3)
- Frail person (SPBB 4-6)
- Pre-frail person (SPBB 7-9)
- Robust person (SPPB 10-12)

RESISTANCE TRAINING

Components	***Exercise details***	***Volume***
**Warm up**	*Initial position:*<br>Sitting on the chair with back straight and feet placed flat on the floor.<br>Standing with feet hip-width apart.<br>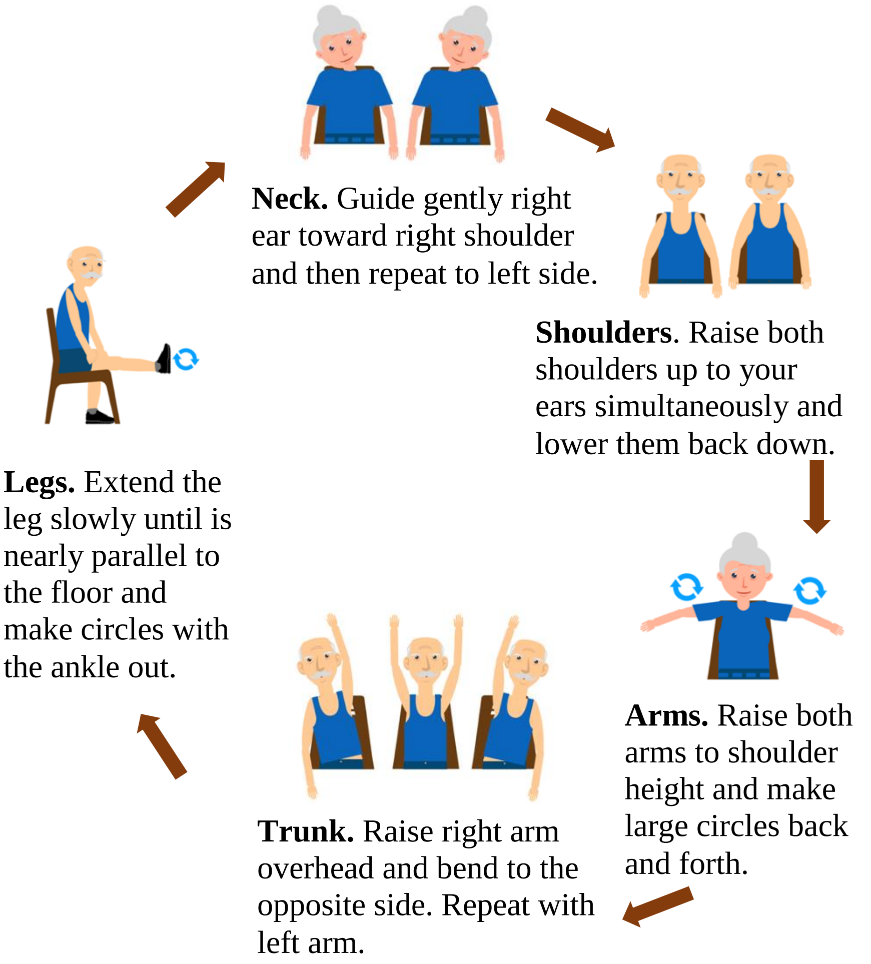	30” per exercise

**Resistance training**	**Lower body limbs**	**Squats.**<br>*Initial position:*<br>Sitting on the chair, with the soles of the feet placed flat on the floor (knees flexed at 90°) and the back straight, resting on the backrest (hip flexed at 90°).<br>*Description:*<br>Get up from the seat as quickly as possible and slowly sit back down.	
		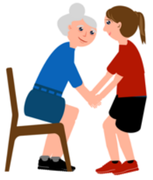 Holding onto the arms of a person standing in front.	1-3 sets,<br>3-5 repetitions<br>1`rest
		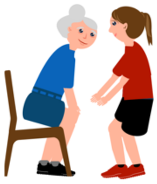 With support on the chair or knees. One person should help to control the movement.	2-3 sets<br>5-8 repetitions<br>1`rest
		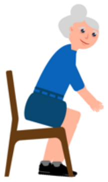 Without help but in a supervised way and without arm support.	2-3 sets,<br>8-10 repetitions<br>1`rest
		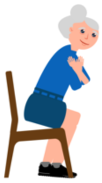 Without help and without arm support. Arms placed crossed on chest.	3 sets,<br>10-12 repetitions<br>1`rest
	**Upper body limbs**	*Initial position:*<br>Sitting on the chair, with the soles of the feet placed flat on the floor (knees flexed at 90°) and the back straight, resting on the backrest (hip flexed at 90°). Patients will hold a bottle in each hand.<br>*Description:*<br>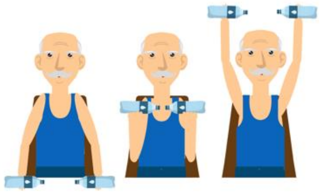 The following arm movements will be performed continuously:<br>1º Raise bottles to chest height, leaving the elbows close to the body.<br>2º Raise arms by stretching them upwards.	1-3 sets,<br>3-5 repetitions<br>(0,25 l)<br>1`rest
			2-3 sets,<br>5-8 repetitions<br>(0,5 l)<br>1`rest
			2-3 sets,<br>10-15repetitions<br>(0,5 l)<br>1`rest
			3 sets,<br>10-12 repetitions<br>(1 l)<br>1`rest

**Balance**	*Initial position:*<br>Standing<br>*Description:*<br>Raise one knee and maintain that position for the indicated time. Turn back to the initial position and repeat with both legs.	
	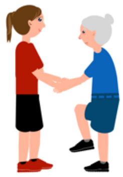 Holding onto a table or another person with both hands.	3 sets,<br>3-5 seconds<br>(open eyes)
	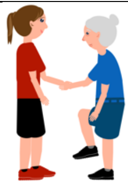 Holding on to a table or another person with one hand. Repeat with closed eyes. 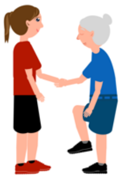	3 sets,<br>3-5 seconds<br>(open and closed eyes)
	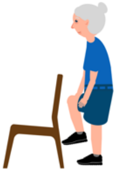 If possible without holding on. Repeat with closed eyes. 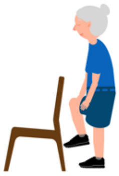	3 sets,<br>3-5 seconds<br>(open and closed eyes)
	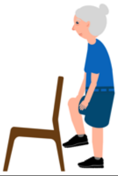 Unassisted but supervised. Repeat with closed eyes. 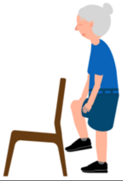	3 sets,<br>5-10 seconds<br>(open and closed eyes)
**Calm down**	*Initial position:*<br>Sitting on the chair.<br>*Description:* Stretches of the main muscle groups.<br>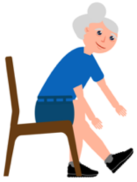<br>**Legs.** Stretch one leg and support the heel. Lean the trunk forward bringing the hands as close to the foot as possible.<br>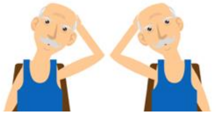<br>**Neck.** Guide gently right ear toward right shoulder with right hand. Stop when you feel a stretch on the left side of neck. Return to neutral. Repeat to left side.<br>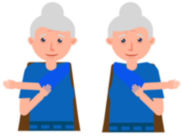<br>**Arms.** Leave your shoulder relaxed and hold your arm above your elbow.<br>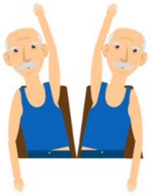<br>**Trunk.** One arm overhead and one arm at side, bend to the side opposite the raised arm.	Keep between 10-15 seconds each exercise

**Inspiratory muscle training**	*Initial position:*<br>Sitting on the chair.<br>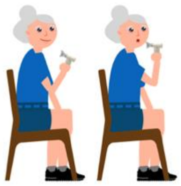<br>*Description:*<br>Perform 30 inspirations through a specific device against 40% of the maximum inspiratory pressure (MIP) that will be measured beforehand and will be adapted to the patient's tolerance.	2 times per day<br>30 repetitions<br>(40% MIP)

ENDURANCE TRAINING

Components	***Description***	***Volume***
**Walking**	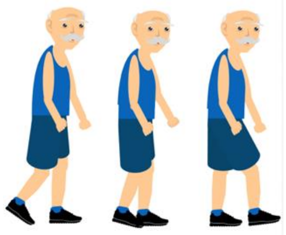<br>-Walk supporting the heel first and then push off with the fingers.<br>-Stand upright, looking straight ahead, and walk at a pace that increases your breathing, speaking should be an effort.<br>-Walk accompanied and/or with support and take a break if needed.	10-15 minutes<br>15-20 minutes<br>20-25 minutes<br>25-30 minutes
**Inspiratory muscle training**	*Initial position:*<br>Sitting on the chair.<br>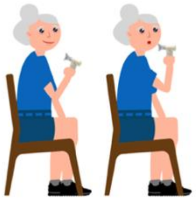<br>*Description:*<br>Perform 30 inspirations through a specific device against 40% of the maximum inspiratory pressure (MIP) that will be measured beforehand and will be adapted to the patient's tolerance.	2 times per day<br>30-35 repetitions<br>(40% MIP)
