# Supplementary material for: Exercise effects on functional capacity and quality of life in older patients with colorectal cancer: study protocol for the ECOOL randomized controlled trial
Source: BMC Geriatr. 2023 May 22;23:314. doi: 10.1186/s12877-023-04026-6 (PMC10201762; doi:10.1186/s12877-023-04026-6)
Supplement: Supplementary file 2 — Additional file 2. [file 12877_2023_4026_MOESM2_ESM.pdf]

Additional file 2

Seated knee extension exercise

Components	***Exercise details***	***Volume***
**Lower body limbs**	*Initial position:*<br>Sitting on the chair with back straight resting on the backrest, the knees bent and feet fully supported on the ground.<br>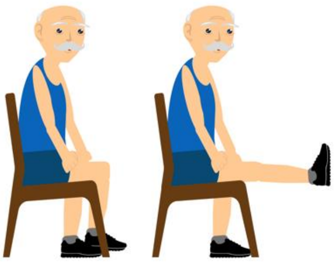<br>*Description*<br>Extend the leg slowly until is nearly parallel to the floor. Try to hold the leg for 2 seconds as straight as possible without locking the knee. Turn back to the initial position and repeat with both legs.	1-3 sets,<br>3-5 repetitions<br>(each leg) / 1`rest<br><br>2-3 sets,<br>5-8 repetitions<br>(each leg) / 1`rest<br><br>2-3 sets,<br>8-10 repetitions<br>(each leg) / 1`rest<br><br>3 sets,<br>10-12 repetitions<br>(each leg) / 1`rest
